# A tale of two metals: Biofortification of rice grains with iron and zinc

**DOI:** 10.3389/fpls.2022.944624

**Published:** 2022-11-07

**Authors:** Andriele Wairich, Felipe K. Ricachenevsky, Sichul Lee

**Affiliations:** ^1^ Graduate Program in Molecular and Cellular Biology, Biotechnology Center, Federal University of Rio Grande do Sul, Porto Alegre, Brazil; ^2^ Department of Botany, Institute of Biosciences, Federal University of Rio Grande do Sul, Porto Alegre, Brazil; ^3^ Center for Plant Aging Research, Institute for Basic Science (IBS), Daegu, South Korea; ^4^ Department of Agricultural Biotechnology, National Institute of Agricultural Science, Jeonju, South Korea

**Keywords:** iron, zinc, rice, biofortification, transporter

## Abstract

Iron (Fe) and zinc (Zn) are essential micronutrients needed by virtually all living organisms, including plants and humans, for proper growth and development. Due to its capacity to easily exchange electrons, Fe is important for electron transport in mitochondria and chloroplasts. Fe is also necessary for chlorophyll synthesis. Zn is a cofactor for several proteins, including Zn-finger transcription factors and redox metabolism enzymes such as copper/Zn superoxide dismutases. In humans, Fe participates in oxygen transport, electron transport, and cell division whereas Zn is involved in nucleic acid metabolism, apoptosis, immunity, and reproduction. Rice (*Oryza sativa* L.) is one of the major staple food crops, feeding over half of the world’s population. However, Fe and Zn concentrations are low in rice grains, especially in the endosperm, which is consumed as white rice. Populations relying heavily on rice and other cereals are prone to Fe and Zn deficiency. One of the most cost-effective solutions to this problem is biofortification, which increases the nutritional value of crops, mainly in their edible organs, without yield reductions. In recent years, several approaches were applied to enhance the accumulation of Fe and Zn in rice seeds, especially in the endosperm. Here, we summarize these attempts involving transgenics and mutant lines, which resulted in Fe and/or Zn biofortification in rice grains. We review rice plant manipulations using ferritin genes, metal transporters, changes in the nicotianamine/phytosiderophore pathway (including biosynthetic genes and transporters), regulators of Fe deficiency responses, and other mutants/overexpressing lines used in gene characterization that resulted in Fe/Zn concentration changes in seeds. This review also discusses research gaps and proposes possible future directions that could be important to increase the concentration and bioavailability of Fe and Zn in rice seeds without the accumulation of deleterious elements. We also emphasize the need for a better understanding of metal homeostasis in rice, the importance of evaluating yield components of plants containing transgenes/mutations under field conditions, and the potential of identifying genes that can be manipulated by gene editing and other nontransgenic approaches.

## Introduction

Iron (Fe) and zinc (Zn) are essential micronutrients for plants and humans. Due to its ability to accept and donate electrons, Fe plays a central role in electron transfer reactions in mitochondria and chloroplasts. Fe is also important for chlorophyll biosynthesis, heme biogenesis, and as enzyme cofactor (Briat et al., 2015). Zn is a necessary cofactor for the activities of enzymes such as Cu/Zn superoxide dismutases, carbonic anhydrase, and alkaline phosphatase (Colvin et al., 2010). Transcription factors, such as Zn-finger domain-containing proteins, also need Zn atoms in their functional structures (Maret, 2009). It is estimated that close to 10% of all *Arabidopsis thaliana* proteins bind Zn (Andreini et al., 2006).

Both elements play roles as cofactors in human proteins (Welch and Graham, 2004). Fe is essential for oxygen transport, because it is part of the functional core of heme group in hemoglobin and myoglobin (Singh et al., 2016). As in plants, Fe is important for electron transport in mitochondria as part of Fe-Sulfur clusters and in DNA replication (Muckenthaler et al., 2017). Zn is important for more than 300 enzymes and 2,000 transcription factors, both structurally and functionally, in humans (Ozyildirim and Baltaci, 2022). Zn is also a signaling molecule in the immune system and in reproduction (Duncan et al., 2016; Singh et al., 2016; Lee et al., 2020; Kim and Lee, 2021).

Rice (*Oryza sativa* L.) is a staple food for more than half of the world’s population, as 19% of all calories, consumed by humans, are derived from rice grain (Elert, 2014). However, rice grain is low in Fe and Zn. In fact, other grains such as wheat and maize are also poor sources of micronutrients, and populations that rely on these grains as staple food suffer from what is known as “hidden hunger” (Alina et al., 2019; Ali, 2021; Stangoulis and Knez, 2022). It is estimated that over 33% of the world’s population are suffering from Fe deficiency anemia (IDA) and up to 20% are Zn deficient (Garcia-Oliveira et al., 2018). Under this scenario, hidden hunger has become a global challenge, with Fe and Zn being two highly prevalent nutritional deficiencies in humans (Rawat et al., 2013; United Nations Environment Programme, 2021). Biofortification, which aims at increasing nutrient concentration in grains and other edible tissues of plants, is a cost-effective approach that can involve conventional plant breeding, genetic engineering, agronomic practices, or combinations of these (Bouis and Saltzman, 2017).

Rice is mostly consumed as white rice, which is dehusked and polished, avoiding that the oil-rich bran layer (rich in oryzanols, tocopherols, tocotrienols, phytosterols, oil and protein, carbohydrate, and dietary fibers) turns rancid at room temperature when stored for a short period of time (Nagendra Prasad et al., 2011). In rice seeds, Fe is accumulated in the dorsal vascular bundle, the aleurone layer scutellum, and the vascular bundle of the embryo scutellum. Zn can penetrate more into the endosperm, but still has uneven distribution, being more concentrated in the outer layers (Takahashi et al., 2009; Johnson et al., 2011; Kappara et al., 2018). Polishing removes both embryo and aleurone, maintaining the starch-rich but Fe- and Zn-poor endosperm (Sperotto et al., 2012a). On average, polished rice of modern high-yielding varieties has concentrations of approximately 2 µg g^-1^ Fe and 16 µg g^-1^ Zn (Trijatmiko et al., 2016). These values are lower than the target values by biofortification programs, 10–15 µg g^-1^ Fe and 28 µg g^-1^ Zn. The addition of Fe and Zn up to these values would provide around 30 and 40% above baseline of the estimated average requirement (EAR) for human diet, as has been used as a reference in the field (Bouis et al., 2011; Mahender et al., 2016). Furthermore, a wide genetic variation for Fe and Zn has been reported for brown rice (dehusked grain without polishing), with concentrations varying from 0.4 to 147 µg g^-1^ and 15.1 to 124 µg g^-1^ for Fe and Zn, respectively (Zeng et al., 2010; Swamy et al., 2021). However, polishing eliminates up to 85% of all Fe, and the observed variability for Fe concentration in polished rice has been found to be limited (Sperotto et al., 2012a; Suman et al., 2021). For Zn, around 25–30% is lost by polishing and a wider genetic variability for Zn concentrations has been observed (Rao et al., 2020). Although eating unpolished/brown rice could at least partially overcome hidden hunger, because it is richer in vitamins and minerals, polished rice is preferred by consumers and has advantages such as longer storage and faster cooking (Liu et al., 2013; Hashimoto et al., 2022). Given that (1) current breeding has not yet achieved the target concentrations for biofortification, (2) genetic engineering strategies showed potential to increase Fe and Zn concentrations, (3) the phenotypes of biofortified rice need to be stable in different environments, and (4) successful biofortification strategies will need to be introduced in local, high-yield genetic backgrounds, it becomes clear that effective biofortification will need a combination of genetic engineering, breeding, and agronomic practices. Therefore, biotechnology has a great potential to improve the concentration of essential nutrients and tackle hidden hunger through biofortification of cereals and other crop plants (Stangoulis and Knez, 2022).

Genetic engineering aiming to increase Fe content in rice endosperm started to be explored more than two decades ago. Common strategies include (1) overexpressing genes involved in Fe uptake from the soil, (2) increasing Fe translocation from roots to shoot and from shoot to grain by manipulating genes involved in synthesis of metal-chelating molecules such as nicotianamine (NA) and mugineic acid (MA), (3) changing expression of genes involved in Fe storage such as ferritin, and (4) manipulating genes that can lower phytic acid content, an anti-nutrient that decreases micronutrient bioavailability (Das et al., 2020). The bottleneck has been the identification of major genes and/or genomic regions that control grain concentrations of Fe, Zn, or both, and their deployment for developing biofortified crops (Gupta et al., 2021), without increasing concentration of harmful elements that can be co-transported with Fe and Zn (Punshon et al., 2018; Ricachenevsky et al., 2018). Although research has advanced substantially in the past decade, the need to deliver biofortified rice grains that can impact human nutrition remains. Moreover, Zn biofortification is largely not addressed, despite the potential for Zn-rich food to positively impact human nutrition worldwide.

The bioavailability of Fe and Zn in rice grain is associated with the concentration of phytic acid (InsP_6_), which is considered the major source of phosphorus and inositol phosphates in cereal grains (White and Broadley, 2009) but is also an antinutrient that reduces Fe^2+^ and Zn^2+^ uptake in the human gut. Multiple studies have aimed to reduce phytate levels in rice grain (Ali et al., 2013; Yamaji et al., 2017; Bhattacharya et al., 2019), a topic that has been thoroughly reviewed elsewhere and falls out of our scope (Perera et al., 2018; Pramitha et al., 2021).

**Figure 1:**
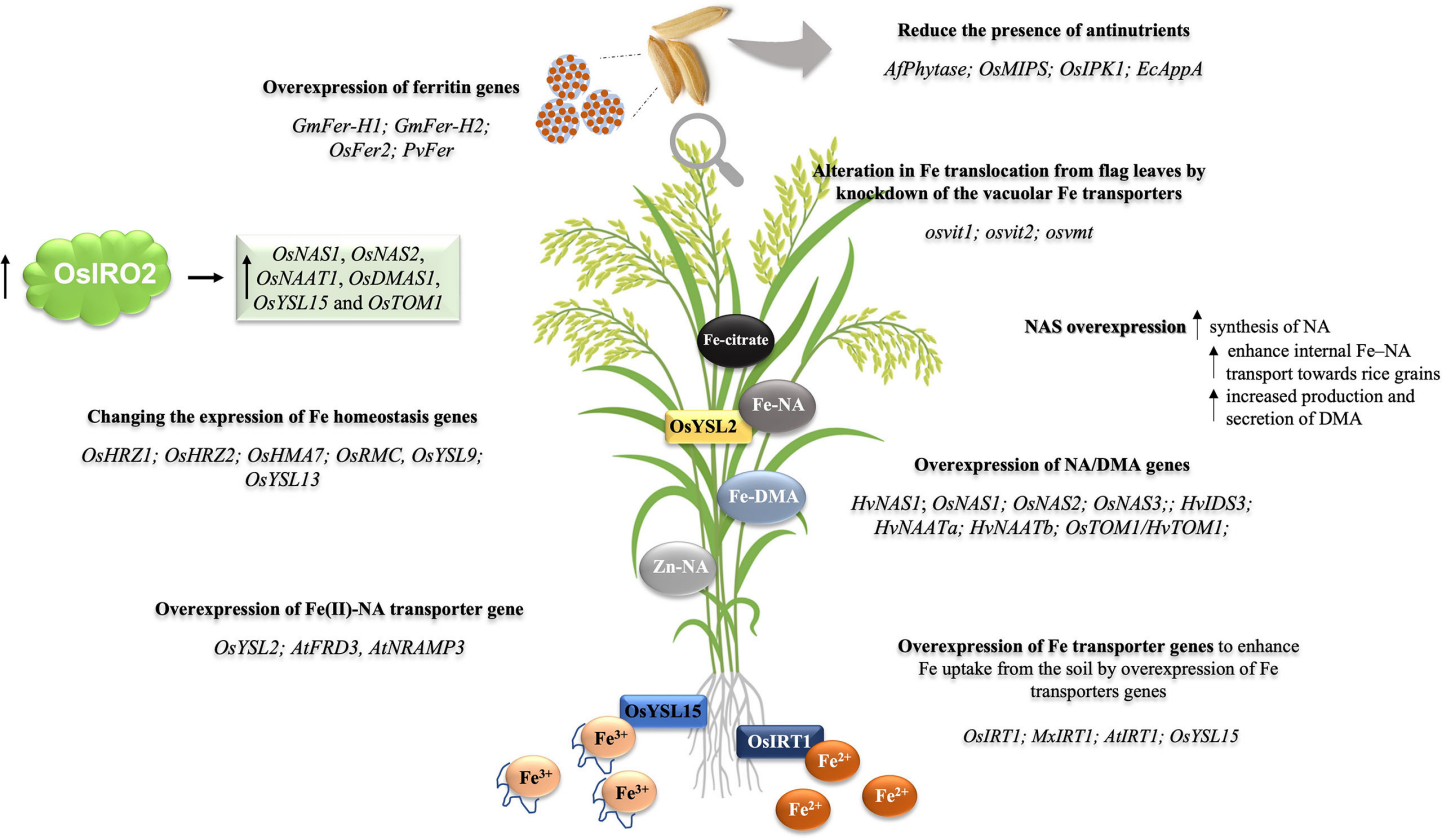
Transgenic approaches to Fe/Zn biofortification in rice plants. The figure presents the main strategies employed for the development of events aiming at higher concentrations of Fe or Fe and Zn in rice grains. (I) The overexpression of ferritin genes, aiming to enhance Fe accumulation in seeds by introducing Fe storage proteins; ferritin genes used: *GmFer-H1*; *GmFer-H2*; *OsFer2*; *PvFer*. (II) Changing the expression of transcriptional regulators of Fe deficiency responses from the bHLH family, OsIRO2, aiming at enhanced Fe uptake and translocation. (III) Changing the expression of Fe homeostasis-related genes. (IV) Enhancing Fe uptake and translocation by the overexpression of Fe transporters, for example, enhance Fe uptake from soil by the overexpression of Fe transporter gene OsIRT1 or OsYSL15. (V) Reduce the presence of antinutrients aiming to enhance the bioavailability of Fe. (VI) Enhancing the translocation from flag leaves to seeds by knockout/knockdown of the vacuolar Fe transporter genes *OsVIT1, OsVIT2*, and *OsVMT*; (VII) Enhancing Fe transport within the plant body by the overexpression of NAS and DMA genes.

Here, we review the genetic engineering approaches for rice biofortification, providing a detailed summary of transgenic/mutant lines generated and the increase in Fe and/or Zn seed concentration in each (**Table 1**). This will allow proper comparisons between approaches and will help researchers decide which ones work best when making new combinations or exploring similar pathways. A graphic representation of these approaches and candidate genes is shown in **Figure 1**. We direct readers to other reviews (Kawakami and Bhullar, 2021), which provide comparative tables in which yield components are evaluated, as well as to reviews where broader aspects of biofortification are discussed (Jha and Warkwntin, 2020; Kamaral et al., 2022; Stangoulis and Knez, 2022; Stanton et al., 2022; Wani et al., 2022).

**Table 1:** List of transgenic/mutant rice approaches to increase rice grain Fe and Zn concentration in seeds.

Approach	Promoter and genes used	Rice genotype	Fe concentration in WT	Zn concentration in WT	Maximum Fe concentration in transgenic	Maximum Zn concentration in transgenic	Fold of Fe increase compared to WT	Fold of Zn increase compared to WT	Reference
Approaches involving transporters and Fe sequestration	*ZmUbi::OsIRT1*	*japonica* cv Dongjin	≈ 11 µg·g^-1^ DW^*^	≈ 20 µg·g^-1^ DW^*^	≈ 12.5 µg·g^-1^ DW^*^	≈ 22 µg·g^-1^ DW^*^	1.13-fold	1.12-fold	(Lee and An, 2009)
	*CaMV 35S::MxIRT1*	NA	≈ 12 µg·g^-1^ DW^*^	≈ 15 µg·g^-1^ DW^*^	30.5 µg·g^-1^ DW^*^	45 µg·g^-1^ DW^*^	2.5-fold^*^	3-fold^*^	(Tan et al., 2015)
	*OsGluB1::GmFer*	*japonica* cv Kitaake	≈ 14.3 µg·g^-1^ DW^*^	–	≈ 38.1 µg·g^-1^ DW^*^	–	2.1-fold	–	(Goto et al., 1999)
	*OsGlu::PvFer* *OsGlu::AfPhytase*	*japonica* cv. Taipei 309	≈ 10.5 µg·g^-1^ DW^*^	–	≈ 23.1 µg·g^-1^ DW^*^	–	2.2-fold^*^	–	(Lucca et al., 2001)
	*OsGluB1::GmFer*	*indica* cv. IR68144	15.7 µg·g^-1^ DW^*^	33.6 µg·g^-1^ DW^*^	≈ 34 µg·g^-1^ DW^*^	≈ 55 µg·g^-1^ DW^*^	1.1 to 2-fold	1.1 to 1.6-fold	(Vasconcelos et al., 2003)
	*OsGluB1::GmFerH1* *OsGluB4::GmFerH1*	*indica* cv. IR64	3.3 µg·g^-1^ DW	–	3.9 µg·g^-1^ DW 7.63.3 µg·g^-1^ DW	–	1.2-fold 2.3-fold	–	(Oliva et al., 2014)
	Activation tag line of *OsNAS3*	*japonica* cv Dongjin	≈ 11 µg·g^-1^ DW^*^ ≈ 4 µg·g^-1^ DW	≈ 20 µg·g^-1^ DW^*^ ≈ 15 µg·g^-1^ DW	≈ 35 µg·g^-1^ DW^*^ ≈ 12 µg·g^-1^ DW	≈ 44 µg·g^-1^ DW^*^ ≈ 35 µg·g^-1^ DW	2.9-fold^*^ 2.6-fold	2.2-fold^*^ 2.2-fold	(Lee et al., 2009b)
	Activation tag line of *OsNAS2 ZmUbi::OsNAS2*	*japonica* cv Dongjin	≈ 9 µg·g^-1^ DW^*^ ≈ 11 µg·g^-1^ DW^*^	–	≈ 27 µg·g^-1^ DW^*^ ≈ 27 µg·g^-1^ DW^*^	–	2.9-fold 2.2 to 2.5-fold	–	(Lee et al., 2012)
	*CaMV35S::OsNAS1* *CaMV35S::OsNAS2* *CaMV35S::OsNAS3*	*japonica* cv. Nipponbare	4.5 µg·g^-1^ DW	≈ 35 µg·g^-1^ DW	56^*^ and 10 µg·g^-1^ DW 81^*^ and 19 µg·g^-1^ DW 63^*^ and 10 µg·g^-1^ DW	59* and 49 µg·g^-1^ DW^*^ 95* and 76 µg·g^-1^ DW 79* and 49 µg·g^-1^ DW	2.4-fold^*^ / 2-fold 3.5-fold^*^ / 4.2-fold 2.7-fold^*^ /2-fold	1.9 -^*^/1.4 -fold 2.5-^*^ /2.2-fold 2.1-^*^/1.4-fold	(Johnson et al., 2011)
	*OsActin1::HvNAS1*	*japonica* cv Tsukinohikari	≈ 1.8 µg·g^-1^ DW	≈ 15 µg·g^-1^ DW	≈ 8 µg·g^-1^ DW	≈ 37.5 µg·g^-1^ DW	4.5-fold	2.5-fold	(Masuda et al., 2009)
	*OsGluB1::OsNAS1*	*japonica* cv Xiushui 110	≈ 12 µg·g^-1^ DW^*^ ≈ 5 µg·g^-1^ DW	≈ 24 µg·g^-1^ DW^*^ ≈ 22 µg·g^-1^ DW	18.68 µg·g^-1^ DW^*^ ≈ 8 µg·g^-1^ DW	36.99 µg·g^-1^ DW^*^ 29.07 µg·g^-1^ DW	1.2 to 1.45-fold^*^	1.3 to 1.6-fold^*^ 1.3-fold	(Zheng et al., 2010)
Further attempts to manipulate the phytosiderophore pathway	point mutation *osnaat1*	*indica* cv Kasalath.	13.7 µg·g^-1^ DW^*^ 1.21 µg·g^-1^ DW	–	24.7 µg·g^-1^ DW^*^ 4.6 µg·g^-1^ DW	–	1.8-fold^*^ 3.8-fold	–	(Cheng et al., 2007)
	*ZmUbi::OsNAS1* + *ZmUbi::HvNAATb*	*O. sativa* cv EYI 105	4 µg·g^-1^ DW	16 µg·g^-1^ DW	16 µg·g^-1^ DW	65 µg·g^-1^ DW	2 to 4-fold	2.2 to 4-fold	(Banakar et al., 2017b)
	*ZmUbi::OsNAS1* + *ZmUbi::HvNAATb* *ZmUbi::OsNAS1* *ZmUbi::HvNAATb*	*O. sativa* cv EYI 105	≈ 20 µg·g^-1^ DW^*^	≈ 21 µg·g^-1^ DW^*^	≈ 58 µg·g^-1^ DW^*^ ≈ 13.4 µg·g^-1^ DW^*^ ≈ 34 µg·g^-1^ DW^*^	≈ 52.5 µg·g^-1^ DW^*^ ≈ 52.5 µg·g^-1^ DW^*^ NA	2.9-fold 0.67-fold 1.7-fold	2.5-fold 1.8 to 2.5-fold NA	(Diaz-Benito et al., 2018)
	*CaMV35S::OsTOM1* *CaMV35S::HvTOM1*	*japonica* cv.Tsukinohikari	≈ 16 µg·g^-1^ DW^*^	≈ 28 µg·g^-1^ DW^*^	≈ 19 µg·g^-1^ DW^*^ ≈ 17 µg·g^-1^ DW^*^	≈ 45 µg·g^-1^ DW^*^ ≈ 32 µg·g^-1^ DW^*^	1.2-fold^*^ 1.2-fold^*^	1.6-fold^*^ 1.2-fold^*^	(Nozoye et al., 2011)
Changing the expression of metal homeostasis-related genes – Fe homeostasis regulators	*OsHRZ1i + OsHRZ2i*	*japonica* cv.Tsukino Hikari	≈2.8 µg·g^-1^ DW	≈28 µg·g^-1^ DW	≈8 µg·g^-1^ DW	≈42 µg·g^-1^ DW	2.9-fold	1.3- to 1.5-fold	(Kobayashi et al., 2013)
	*CaMV35S::OsIMA1* *CaMV35S::OsIMA2*	*japonica* cv.Tsukino Hikari	≈18 µg·g^-1^ DW^*^	≈35 µg·g^-1^ DW^*^	≈60 µg·g^-1^ DW^*^ ≈75 µg·g^-1^ DW^*^	≈50 µg·g^-1^ DW^*^ ≈60 µg·g^-1^ DW^*^	2.5 to 3.4-fold^*^ 2.3- to 4.2-fold^*^	1.4-fold^*^ 1.3- to 1.7-fold^*^	(Kobayashi et al., 2021)
	*CaMV35S::OsIRO2*	*japonica* cv.Tsukino Hikari	≈6 µg·g^-1^ DW	–	≈16 µg·g^-1^ DW	–	2.6-fold	–	(Ogo et al., 2011)
	*CaMV35S::OsbHLH058*	*japonica* cv.Tsukino Hikari	≈16 µg·g^-1^ DW^*^	–	≈32 µg·g^-1^ DW^*^	–	1.8-fold^*^	–	(Kobayashi et al., 2019)
	*OsbHLH059i*	*japonica* cv.Tsukino Hikari	≈16 µg·g^-1^ DW^*^	–	≈20 µg·g^-1^ DW^*^	–	1.2-fold^*^	–	(Kobayashi et al., 2019)
	Ubi::OHMA7 allele 261^***^ *Ubi::OHMA7 allele 284*	*japonica* cv Kitaake	≈12 µg·g^-1^ DW^*^	≈33 µg·g^-1^ DW^*^	≈60 µg·g^-1^ DW^*^ ≈100 µg·g^-1^ DW^*^	≈80 µg·g^-1^ DW^*^ ≈112 µg·g^-1^ DW^*^	5-fold 8.2-fold	2.5-fold 3.5-fold	(Kappara et al., 2018)
Changing the expression of metal homeostasis-related genes - Fe homeostasis transporters and binding molecules	T-DNA insertion mutant line: *osvit1*	*japonica* cv. Zhonghua 11	≈22 µg·g^-1^ DW^*^	≈42 µg·g^-1^ DW^*^	≈28 µg·g^-1^ DW^*^	≈50 µg·g^-1^ DW^*^	1.3-fold^*^	1.2-fold^*^	(Zhang et al., 2012)
	T-DNA insertion mutant line: *osvit2*	*japonica* cv. Dongjin	≈5 µg·g^-1^ DW	≈23 µg·g^-1^ DW	≈9 µg·g^-1^ DW	≈36 µg·g^-1^ DW	1.4-fold^*^/1.8-fold	1.6-fold	(Zhang et al., 2012 / Bashir et al., 2013)
	Knockout lines by CRISPR/Cas9 *osvmt*	*japonica* cv. Nipponbare	≈4.2µg·g^-1^ DW	≈22 µg·g^-1^ DW	≈8.4 µg·g^-1^ DW	≈35 µg·g^-1^ DW	2-fold	1.6-fold	(Che et al., 2019)
	*OsActin::OsYSL15*	*japonica* cv. Dongjin	≈12 µg·g^-1^ DW^*^	–	≈14.5 µg·g^-1^ DW^*^	–	1.25-fold^*^	–	(Lee et al., 2009a)
	*OsSUT1::OsYSL2*	*japonica* cv.Tsukino Hikari	≈1.8 µg·g^-1^ DW	–	≈8 µg·g^-1^ DW	–	4.4-fold	–	(Ishimaru et al., 2010)
	*osysl9i*	*japonica* cv.Tsukino Hikari	≈1 µg·g^-1^ DW	–	≈2.5 µg·g^-1^ DW	–	2.5-fold	–	(Senoura et al., 2017)
	*CaMV35S::OsYSL13*	*japonica* cv. Nipponbare	≈21 µg·g^-1^ DW^*^	–	≈26 µg·g^-1^ DW^*^	–	1.25-fold^*^	–	(Zhang et al., 2018)
	*ZmUbi::HvYS1*	*O. sativa* cv EYI 105	≈4 µg·g^-1^ DW	–	8.7 µg·g^-1^ DW	–	2.1-fold	–	(Banakar et al., 2017a)
	*osmit*	*japonica* cv. Dongjin	–	≈23 µg·g^-1^ DW	–	≈36 µg·g^-1^ DW	–	1.6-fold	(Bashir et al., 2013)
Other approaches that resulted in increased Fe concentration	*CaMV35S::AtCOPT1-*	*japonica* cv. Nipponbare	≈ 17 µg·g^-1^ DW^*^ ≈ 5 µg·g^-1^ DW	–	≈ 22 µg·g^-1^ DW^*^ ≈ 8 µg·g^-1^ DW	–	1.3-fold^*^ 1.6-fold	–	(Andrés-Bordería et al., 2017)
	*CtCBM-IBP*	*japonica* cv. Nipponbare	≈ 2 µg·g^-1^ DW	–	≈ 6 µg·g^-1^ DW	–	3-fold	–	(Yang et al., 2016)
	*CaMV35S::OsRMC* *osrmci*	*japonica* cv. Zhonghua *10*					1.09-fold 0.83-fold	1.2-fold 0.9-fold	(Yang et al., 2013)
	*OsUbi::OsRab6a*	*japonica* cv. Zhonghua 10	≈22 µg·g^-1^ DW^*^	–	≈25 µg·g^-1^ DW^*^	–	1.1 to 1.25-fold^*^	–	(Yang et al., 2020a)
Zn biofortification- still lacking basic knowledge	*CaMV35S::OsZIP9*	*japonica* cv Nipponbare	–	≈25 µg·g^-1^ DW		≈33 µg·g^-1^ DW	–	1.3-fold	(Tan et al., 2020)
	*osmt2b* *osmt2c* *osmt2bosmt2c*	*japonica* cv Nipponbare *japonica* cv Dongjin	–	≈45 µg·g^-1^ DW^*^ ≈35 µg·g^-1^ DW^*^	–	≈36 µg·g^-1^ DW^*^ ≈38 µg·g^-1^ DW^*^ ≈25 µg·g^-1^ DW^*^	–	0.8-fold^*^ 0.8-fold^*^ 0.7-fold^*^	(Lei et al., 2021)
	*dmas*	*japonica* cv Dongjin	–	≈18 µg·g^-1^ DW	–	≈22 µg·g^-1^ DW	–	1.2-fold	(Bashir et al., 2017)
Combination of approaches	*CaMV35S::OsNAS2 + GluA2::GmFer-H1*	*indica* cv. IR64	≈ 2 µg·g^-1^ DW	≈ 12 µg·g^-1^ DW	15 µg·g^-1^ DW	45.7 µg·g^-1^ DW	7.5-fold	2.7- to 3.8-fold	(Trijatmiko et al., 2016)
	*OsGluB1::GmFer-H2 + OsGluB1::OsYSL2 + OsGluB1:: GmFer-H2 + OsActin1::HvNAS1 + OsSUT1::OsYSL2*	Tropical *japonica* cv. Paw San Yin	1.5 µg·g^-1^ DW	29.5 µg·g^-1^ DW	5.02 µg·g^-1^ DW	39.2 µg·g^-1^ DW	3.4-fold	1.3-fold	(Aung et al., 2013)
	*OsGlb:: GmFerH2 + OsGluB::HvNAAT-A + OsGluB::HvNAS1 + OsGluB1::GmFerH2 + OsGluB1::HvIDS3*	*japonica* cv.Tsukino Hikari	1.1 µg·g^-1^ DW	≈ 22 µg·g^-1^ DW	4.9 µg·g^-1^ DW	≈ 30 µg·g^-1^ DW	2.5 to 4-fold	1.35-fold	(Masuda et al., 2013)
	*OsGlB::PvFer + OsGlB::AfPhytase + CaMV35S::AtNAS*	*japonica* cv. Taipei 309	≈1 µg·g^-1^ DW	≈ 20 µg·g^-1^ DW	≈6.3 µg·g^-1^ DW	≈ 32 µg·g^-1^ DW	6.3- fold	1.3 to 1.5-fold	(Wirth et al., 2009)
	*MsEnod12B::AtIRT1*	Lines developed by Wirth et al. (2009)	4 µg·g^-1^ DW	≈30 µg·g^-1^ DW	9.6 µg·g^-1^ DW	≈45 µg·g^-1^ DW	2.2-fold	1.5-fold	(Boonyaves et al., 2016)
	*MsEnod12B::AtIRT1 + OsGluB1::PvFER + CaMV35S::OsNAS1* *AtIRT1::AtIRT1 + OsGluB1::PvFER + CaMV35S::OsNAS1*	*japonica* cv. Nipponbare	2.5 µg·g^-1^ DW	≈18 µg·g^-1^ DW	≈ 4.5 µg·g^-1^ DW ≈9.5 µg·g^-1^ DW	NA ≈32.4 µg·g^-1^ DW	1.8-fold 3.8-fold	NA 1.8-fold	(Boonyaves et al., 2017)
	*OsGlB1::PvFER + CaMV35S::AtNAS1 + ZmUbi::AtFRD3* *OsGlB1::PvFER + ZmUbi::AtFRD3*	*japonica* cv. Nipponbare	≈2.5 µg·g^-1^ DW	≈17 µg·g^-1^ DW	≈13.25 µg·g^-1^ DW	≈41 µg·g^-1^ DW ≈29 µg·g^-1^ DW	3.3 to 5.3-fold^**^	2.44-fold 1.7-fold	(Wu et al., 2018)
	*CaMV35S::AtNAS1 + OsGlB1::PvFER + ZmUbi::AtNRAMP3* *CaMV35S::AtNAS1 + OsGlB1::PvFER + OsOle18::AtNRAMP3* *OsGlB1::PvFER + ZmUbi::AtNRAMP3* *OsGlB1::PvFER +OsOle18::AtNRAMP3*	*japonica* cv. Nipponbare	≈2.5 µg·g^-1^ DW	≈20 µg·g^-1^ DW	≈15 µg·g^-1^ DW	≈48 µg·g^-1^ DW	3.6- to 6-fold^**^	1.5 to 2.4-fold^**^	(Wu et al., 2019)
	*OsHMA2::ZmYS1* *OsFRDL1:OsTOM1* *OsHMA2::ZmYS1* + *OsFRDL1:OsTOM1* *OsHMA2::ZmYS1* + *OsFRDL1:OsTOM1* + *OsGlob1:GmFer H1* *OsHMA2::ZmYS1* + *OsFRDL1:OsTOM1* + *OsGlob1:GmFer H1* + ZmUbq1::HvNAS1	*japonica* cv. Nipponbare	≈1.5 µg·g^-1^ DW	≈30 µg·g^-1^ DW	≈7 µg·g^-1^ DW ≈5 µg·g^-1^ DW - ≈13 µg·g^-1^ DW ≈14 µg·g^-1^ DW	≈45 µg·g^-1^ DW ≈36 µg·g^-1^ DW ≈39 µg·g^-1^ DW ≈42 µg·g^-1^ DW ≈36 µg·g^-1^ DW	4.8-fold 3.2-fold - 8.7-fold 9.3-fold	1.5-fold 1.2-fold 1.3-fold 1.4-fold 1.2-fold	(Kawakami et al., 2022)

*unpolished seeds. **average for all constructs. ***transgenic lines did not survive or yield enough seeds.

## Manipulations of Fe transporters and Fe storage

Surprisingly, attempts at biofortification involving Fe and Zn transporter manipulation, either changing expression patterns or substrate specificity, are scarce, which may derive from the lack of literature on how plants deliver Fe and Zn to seeds, especially in grasses. One of the first examples was the overexpression of rice *Iron-Regulated Transporter 1* (*OsIRT1*) under the control of *ZmUbi* (Lee and An, 2009). Overexpression of *OsIRT1* slightly increased Fe and Zn concentrations in brown seeds compared with WT plants (1.13- and 1.12-fold, respectively). However, transgenic plants were shorter and had fewer tillers, probably as a consequence of disturbed metal homeostasis (Lee and An, 2009). Following a similar approach, *MxIRT1* (from *Malus xiaojinensis*) was overexpressed in rice, leading to a threefold increase of Fe and Zn in brown seeds but without significant changes in plant size. However, authors have not reported whether tillering or seed set was altered (Tan et al., 2015).

Conversely, the manipulation of Fe storage or sequestration by changing *Ferritin* expression is one of first ideas attempted for Fe biofortification. Ferritin is an Fe-storage protein found in plants, animals, and bacteria. Plant ferritin is estimated to accumulate up to 4,500 atoms of Fe in a bioavailable form (Briat et al., 2010). Approaches to induce Fe accumulation in rice seeds by tissue-specific expression of *ferritin* genes were successful to some extent. Early work using soybean (*Glycine max*) *ferritin* gene (*GmFerH1*) driven by the endosperm-specific *OsGluB1* (Glutelin B1, LOC_Os02g15169 / Os02g0249900; Masuda et al., 2013) promoter in the rice Kitaake background (Goto et al., 1999) resulted in threefold higher Fe levels in brown rice of transgenic lines, ranging from 13.3 to 38.1 µg g^-1^, whereas in WT varied from 8.6 to 14.3 µg g^-1^. In polished rice, Fe concentrations averaged 3.4 µg g^-1^ in grains from the transgenic lines, whereas those in WT averaged 1.6 µg g^-1^ (Goto et al., 1999).

Similar approaches were used in distinct genetic backgrounds, with similar results. *GmFer* driven by the endosperm-specific *OsGluB1* promoter was expressed in the *indica* elite rice line IR68144 (Gregorio et al., 2000). Fe concentration in polished rice seeds ranged from 17 to 34 µg g^-1^ compared with 15 µg g^-1^ in WT seeds. Zn levels also increased, ranging from 36 to 55 µg g^-1^ compared with 33 µg g^-1^ in WT (Vasconcelos et al., 2003). Similarly, the expression of *ferritin* from lima bean (*Phaseolus limensis*) under the control of *GluB1* promoter in the Chinese high-yield variety Wuxiangjin 9 led to 64% increase in Fe concentration in polished rice compared with WT (Liu et al., 2004). The expression of a *ferritin* gene from *Phaseolus vulgaris* driven by an endosperm-specific promoter in Taipei 309 background resulted in increased Fe concentration in brown rice, ranged from 11.5 to 22 µg g^-1^, a twofold increase compared with WT (9.9–10.65 µg g^-1^ Fe) (Lucca et al., 2001).

Therefore, the endosperm-specific expression of *ferritin* resulted in increased Fe in polished rice grain. However, despite the increase in ferritin protein in the endosperm, Fe loading seems to be limited, because transgenic lines with six- to sevenfold increase in ferritin protein expression were shown to achieve only approximately threefold increase in grain Fe concentration (Oliva et al., 2014). This suggests that ferritin accumulation does not lead to a proportional increase in Fe concentration. Therefore, other bottlenecks may exist and need to be overcome to further increase Fe loading in the endosperm. Still, these works show that *ferritin* expression in endosperm is useful for biofortification and that the use of other genes to manipulate Fe allocation to the endosperm might be a promising alternative.

## Strategic manipulation of nicotianamine synthase, phytosiderophore synthesis and transport genes in iron and zinc biofortification

Rice is a plant that uses the “Combined Strategy” for Fe uptake, which means that it has a full chelation strategy and also uses the Fe(II) transporter OsIRT1 for Fe uptake (Ishimaru et al., 2006; Wairich et al., 2019; Pradhan et al., 2020; Bashir and Ishimaru, 2021). The chelation strategy is based on phytosiderophore (PS) release to the rhizosphere. In rice, the PS 2′-deoxymugineic acid (DMA) is secreted into the rhizosphere through OsTOM1 (*Transporter of Mugineic acid family*)/OsZIFL4 (*Zinc-Induced Facilitator-Like*) transporter (Nozoye et al., 2011; Ricachenevsky et al., 2011). In the rhizosphere, DMA chelates Fe(III) and the complex Fe(III)-DMA is transported into root cells by the transmembrane protein Yellow Stripe-like 15 (OsYSL15) (Inoue et al., 2009; Lee et al 2009a). Rice plants can also directly uptake Fe(II) by OsIRT1/OsIRT2 transporters (Ishimaru et al., 2006), a trait that is shared with *Oryza* species from the *Oryza sativa* complex (AA genome; Wairich et al., 2019). This could be an adaptation to habitat changes, such as from upland to deep water (Menguer et al., 2017), where Fe(II) is more available, similar to flooded soils in which domesticated rice is cultivated nowadays.

Interestingly, DMA and its precursor, NA are also involved in long-distance metal transport within the plant. NA is biosynthesized by trimerization of S-adenosylmethionine by NA synthase (NAS) enzymes, which is then converted into a 3′′-keto intermediate by NA amino transferase (NAAT) (Takahashi et al., 1999), and next converted into DMA by the reduction of 3′′-carbon of the keto intermediate by deoxymugineic acid synthase (DMAS) (Bashir et al., 2006). NA chelates Fe(II) and Zn(II) and is involved in transport of both metals in the phloem (Nozoye, 2018). Given that phloem translocation is involved in delivering Fe and Zn to developing seeds (Sperotto et al., 2012b), manipulation of *NAS* expression has been explored for biofortification.

The rice genome encodes three *NAS* genes, *OsNAS1*, *OsNAS2*, and *OsNAS3*, which are differentially regulated by Fe (Inoue et al., 2003). In a study with *OsNAS3* activation-tagged mutant, Lee et al. (2009b) showed that unpolished grain from mutants had higher concentrations of Fe (2.9-fold), Zn (2.2-fold), and Cu (1.7-fold) than WT plants. Similar increases in Fe (2.6-fold) and Zn (2.2-fold) concentrations were observed in polished rice, whereas NA and DMA concentration in seeds were increased 9.6- and 4.0-fold, respectively (Lee et al., 2009b). Similarly, *OsNAS2* activation-tagged mutants and OE-*OsNAS2* transgenic plants driven by the maize *ubiquitin* (*ZmUbi*) promoter increased Fe concentration in brown rice by 2.3- to 3.0-fold, whereas Fe concentrations in polished rice were 2.2 to 2.9-fold higher, comparing transgenic lines to WT (Lee et al., 2012). Importantly, mice fed with seeds from both activation-tagged mutant lines recovered more rapidly from dietary Fe and Zn deficiency than mice fed with WT seeds, suggesting both nutrients become more bioavailable by increased NA concentration (Lee et al., 2009b; Lee et al., 2011; Lee et al., 2012; **Table 1** and **Figure 1**).

In an independent work, the overexpression of *OsNAS1*, *OsNAS2*, or *OsNAS3* under the control of the constitutive promoter *CaMV35S* in rice plants also resulted in increased metal concentrations (Johnson et al., 2011). Brown rice Fe concentrations were 2.4-, 3.5-, and 2.7-fold higher in OE*-OsNAS1*, OE*-OsNAS2*, and OE*-OsNAS3*, respectively, compared with WT, whereas Zn concentrations were 1.9-, 2.5-, and 2.1-fold higher, respectively. Fe and Zn concentrations were also increased in polished seeds, especially in *OsNAS2*-overexpressing lines, which achieved up to 4.2-fold for Fe and 2.2-fold for Zn. These increases were positively correlated with NA concentration and are well within the target range for biofortification (Johnson et al., 2011; Moreno-Moyano et al., 2016). In a work using heterologous expression, rice transgenic lines expressing barley (*Hordeum vulgare*) *HvNAS1* under the control of *OsActin1* promoter were shown to have increased Fe and Zn concentrations in polished seeds by 4.5- and 2.5-fold, respectively, compared with non-transformed plants (Masuda et al., 2009). Besides the variation in fold-change numbers, these studies demonstrated that *NAS* overexpression in rice results in increased Fe and Zn concentration in seeds.

In a different approach using *NAS* genes, an elite rice genotype from China expressing *OsNAS1* under the control of *OsGluB1* promoter reached significant increase in NA concentration in grain, leading to increased concentration of Fe and Zn in brown rice. However, the Fe concentrations in polished grain were similar to WT (Zheng et al., 2010). Still, *in vitro* Caco-2 cell digestion model showed that Fe from transgenic polished grain was more bioavailable (2- to 2.3-fold), suggesting that NA has a role in increasing Fe bioavailability independently from its role in increasing metal concentration. Zn concentration, on the other hand, increased significantly in these lines (~1.5-fold) in both unpolished and polished grain (Zheng et al., 2010). Taken together, these studies established that manipulation of *NAS* gene expression enhances internal Fe– and Zn–NA translocation to rice grain and showed that manipulation of NA levels in rice plants is a feasible strategy for biofortification.

However, it should be noted that changes in NA concentrations in rice also resulted in decreased yield in some studies (Moreno-Moyano et al., 2016; Trijatmiko et al., 2016; for a review, see Kawakami and Bhullar, 2021), suggesting that there are trade-offs in NA overaccumulation. Therefore, future work should focus on fine tuning of *NAS* expression. For example, NA accumulation in phloem cells is important for biofortification and should be further explored using specific promoters. On the other hand, NA overaccumulation in specific organelles, such as chloroplast and mitochondria, could be detrimental, and should also be addressed (Lee et al., 2009b; Lee et al., 2012). Moreover, despite the known relationship of *NAS* genes with Fe homeostasis, there is not a clear understanding of the role of each *NAS* in rice. Functional characterization of *NAS* genes using single and higher order mutants, as done in *A. thaliana* (Klatte et al., 2009; Schuler et al., 2012), may shed light on the physiological nature of these trade-offs, which could explain the yield effects observed (Kawakami and Bhullar, 2021) and provide knowledge to use *NAS* as a tool for biofortification.

A knockout mutation in the gene encoding the PS synthesis enzyme *OsNAAT1* led to disrupted DMA biosynthesis. When these lines were cultivated in field conditions under continuous flooding, they grew normally and accumulated more Fe in seeds than WT plants. The interpretation was that, although *osnaat1* loss of function precluded DMA secretion and Fe(III) uptake through OsYSL15, Fe(II) uptake was still functional, allowing growth under flooded conditions (Cheng et al., 2007). It also raised the question whether plants relying only on Fe(II) uptake achieve higher Fe concentrations in seeds. Fe concentrations in unpolished and polished grain were 1.8- and 3.8-fold higher than their WT, respectively (Cheng et al., 2007). Banakar et al. (2017b) produced transgenic lines overexpressing rice *OsNAS1* and barley *HvNAATb* under the control of *ZmUbi*. Endosperm Fe levels in these lines were 1.4–3.7 times higher than in WT, and Zn levels were also boosted by 1.2- to 4.2-fold, while limiting the accumulation of cadmium (Cd), a harmful element that can often co-accumulate in rice endosperm, demonstrating that manipulation of the same pathway beyond *NAS* genes might be beneficial (Banakar et al., 2017b). Importantly, the authors suggest that there is an upper limit for Fe (~22.5 µg g^-1^) and Zn (~84 µg g^-1^) accumulation in the endosperm, regardless of the external Fe concentration in soil solution. This upper limit occurs possibly as a homeostatic mechanism to prevent Fe overloading during seed development, and these values never exceeded 4.6-fold more than the value observed in WT (Banakar et al., 2017b), which may represent a trade-off to biofortification. Additionally, these values are consistent with the limited natural genetic diversity of Fe levels in rice, as was observed ranging from 2 to 5 µg g^-1^ DW (Sperotto et al., 2012b). A threshold of 6 µg Fe g^−1^ DW in plants expressing *AtNAS1* was observed by Wirth et al. (2009), and a threshold of 14 µg Fe g^−1^ DW in plants expressing *OsNAS2* was observed by Johnson et al. (2011). The results described above highlight how little we know about the mechanisms orchestrating Fe and Zn accumulation in rice seeds.

Rice plants overexpressing *OsNAS1* and/or *HvNAATb* were generated to evaluate whether contrasting levels of NA and DMA would play a role in Fe and Zn distribution in embryo and endosperm of rice seeds. The endosperm Fe concentrations in *OsNAS1* and *OsNAS1 + HvNAATb* co-transformed lines were 1.7- to 2.9-fold higher than in WT plants. On the other hand, the Fe concentrations were 33% lower in the endosperm of *HvNAATb*-only and co-transformed lines. Additionally, endosperm Zn concentrations in *OsNAS1* and *OsNAS1 + HvNAATb* co-transformed lines increased by 1.8- to 2.5-fold and 2.5-fold, respectively (Diaz-Benito et al., 2018). Taken together, these results demonstrate that changing NA/DMA concentrations by fine tuning the expression of biosynthetic genes is a promising avenue to deliver biofortified rice. However, we still need to clearly understand the physiological roles of each gene involved in the PS synthesis pathway and the precise roles of NA/DMA in rice plants.

An alternative approach to achieve Fe biofortification by manipulating PSs was the overexpression of the OsTOM1/OsZIFL4 transporter, which is directly involved in the efflux of DMA from the roots to the rhizosphere (Nozoye et al., 2011). *OsTOM1* is part of the *ZIFL* gene family that has expanded in grasses probably as a result of larger use of PSs (Ricachenevsky et al., 2011). Rice plants overexpressing either *OsTOM1* or *HvTOM1* driven by *CaMV35S* promoter have increased DMA secretion from roots and improved tolerance to Fe deficiency, as well as a 1.2-fold increase in brown seed Fe and Zn concentrations compared with WT plants (Nozoye et al., 2011). It is not clear, however, how the increased expression of this transporter leads to changes in seed concentration, and more studies are necessary to uncover other possible roles of *OsTOM1/OsZIFL4* in metal accumulation in seeds.

## Changing the expression of metal homeostasis-related genes

Work focused on understanding the biological role of Fe and Zn homeostasis often produce plants with increased concentrations of Fe or Zn. Although these studies were not attempted to generate biofortified plants *per se*, they inform the community about good targets and mechanisms that can be explored for biofortification. Here, we review these studies, which can be useful for devising new single and multi-gene biofortification strategies. These data are also summarized in **Table 1** and **Figure 1**.

*Hemerythrin motif-containing Really interesting new gene- and Zinc-finger proteins 1* and *2* (*OsHRZ1* and *OsHRZ2*) are homologous to the *E3 ubiquitin ligases BRUTUS/BRUTUS-Like* from *A. thaliana* (Selote et al., 2015; Hindt et al., 2017; Rodríguez-Celma et al., 2019). These proteins act as negative regulators of the Fe deficiency response, degrading key components involved in increasing Fe uptake by roots. *OsHRZ1* and *OsHRZ2* play a similar role as negative regulators of Fe deficiency-inducible genes for Fe uptake and translocation in rice roots and are also proposed to function as Fe sensors (Kobayashi et al., 2013). Rice plants knocked down for *OsHRZ1* and *OsHRZ2* grew healthily and without visible phenotype under normal conditions and accumulated up to 2.9-fold more Fe in polished seeds and up to 1.3- to 1.5-fold Zn compared with WT (Kobayashi et al., 2013). OsHRZs were later shown to be involved in a reciprocal regulatory module linking Fe and phosphorus homeostasis (Guo et al., 2021), suggesting that manipulation of these genes for biofortification, although possible, can have pleiotropic effects. Therefore, aiming at regulators such as HRZ might have deleterious effects and understanding of its functional in integrating multiple signals should be a focus in the field.

IRON MAN (IMA) proteins were first described in *A. thaliana* as positive regulators of Fe-deficiency-inducible genes for Fe uptake (Grillet et al., 2018). In rice, two *IMA* genes (*OsIMA1* and *OsIMA2*) were shown to be involved in modulating the Fe deficiency response (Kobayashi et al., 2021). Transgenic rice lines overexpressing *OsIMA1* and *OsIMA2* under the control of *CaMV35S* promoter increased Fe concentration in brown rice from 2.5- to 3.4-fold in OE-*OsIMA1* and from 2.3- to 4.2-fold in OE-*OsIMA2*. Likewise, the Zn concentration also increases 1.4-fold in OE-*OsIMA1* and from 1.3- to 1.7-fold in OE-*OsIMA2*. Additionally, these lines were more tolerant to Fe deficiency than WT plants (Kobayashi et al., 2021). As overexpression of *OsIMA* genes and knockdown of *HRZs* confer similar phenotypes (Kobayashi et al., 2013; Kobayashi et al., 2021), a promising strategy to enhance Fe and Zn concentrations in rice grain might rely on simultaneously altering the expression of members of both gene families.

bHLH (basic Helix–Loop–Helix) proteins play a role as transcriptional regulators of Fe deficiency responses in plants (reviewed by Gao and Dubos, 2021; Riaz et al., 2021). *OsIRO2*, an Fe-deficiency inducible *bHLH* transcription factor, positively regulates genes involved in Fe deficiency response, such as *OsNAS1*, *OsNAS2*, *OsNAAT1*, *OsDMAS1*, *OsYSL15*, and *OsTOM1* (Ogo et al., 2006). Plants overexpressing *OsIRO2* displayed increased tolerance to low-Fe availability in long-term cultivation and grew better than WT plants when grown on calcareous soil. Additionally, OE-*OsIRO2* lines accumulated twofold higher Fe in grain than WT when cultivated in calcareous soil (Ogo et al., 2011). The *bHLH* transcription factors subgroup IVc *OsbHLH058*, and *OsbHLH059* are also associated to Fe deficiency responses and are downregulated under Fe deficiency (Kobayashi et al., 2019). Transgenic lines overexpressing or knockdown for *OsbHLH058* and *OsbHLH059* were developed. OE*-OsbHLH058* lines had improved tolerance to low Fe when compared with WT while also having significantly higher Fe concentration in brown seeds (~1.8-fold). On the other hand, *osbHLH058-i* and *osbHLH059-i* knockdown lines were more susceptible to low Fe availability, and brown seeds of *osbHLH058-i* had approximately 20% less Fe compared with WT lines. Counterintuitively, brown seeds from *osbHLH059-i* lines had 1.2-fold increase in Fe concentrations (Kobayashi et al., 2019). These results show that manipulation of regulatory proteins that control multiple genes, such as transcription factors protein, stability regulators, and others, should be taken cautiously when aiming at biofortification, because they may lead to undesirable pleiotropic effects.

OsHMA7 is a transporter from the Heavy Metal Associated family, and its function was uncharacterized until recently. Two *OsHMA7* alleles from contrasting recombinant introgression lines (261 and 284 with high- and low-grain Fe and Zn concentrations, respectively) were identified (Kappara et al., 2018). The overexpression of the 261 (OE-261) allele resulted in increased grain concentrations of Fe (up to 60 µg g^-1^) and Zn (up to 80 µg g^-1^) in the T0 generation. However, the transgenic lines did not survive or yield enough seeds. On the other hand, the OE-284 line resulted in increases of Fe (up to 99.07 µg g^-1^) and Zn (up to 112.5 µg g^-1^) concentrations. These values are significantly higher than those observed in non-transformed Kitaake plants (12–13 µg g^-1^ for Fe and 32–33 µg g^-1^ for Zn, respectively). In addition, the OE lines acquired tolerance to Fe- and Zn-deficient soils. The transcript level of *OsHMA7* was fivefold higher in OE-284 lines than in Kitaake, a change that altered transcript levels of Fe- and Zn-responsive genes (Kappara et al., 2018). These results suggest that OsHMA7 may play a role in regulating Fe and Zn concentrations in rice grain.

Transporters associated with regulation of Fe traffic between cytosol and vacuole have been identified in *A. thaliana* and rice (Kim et al., 2006; Zhang et al., 2012). Arabidopsis *Vacuolar Iron Transporter 1* (*AtVIT1*) mediates vacuolar sequestration of Fe^2+^ and Mn^2+^ and is highly expressed in developing seeds. Interestingly, seeds from *atvit1* plants have similar Fe concentrations compared with WT, whereas spatial distribution is severely altered, resulting in lower germination in alkaline soil (Kim et al., 2006). Rice homologous genes *OsVIT1* and *OsVIT2* were characterized: Both are highly expressed in flag leaves, whereas the encoded proteins transport Fe and Zn into vacuoles. Disruption of *OsVIT1* and *OsVIT2* leads to increased accumulation of Fe and Zn in brown rice seeds (1.3- to 1.4-fold for Fe and 1.2- to 1.6-fold for Zn), whereas flag leaf concentrations are decreased (Zhang et al., 2012). In polished *osvit2* seeds, the concentrations of Fe and Zn are significantly higher (Bashir et al., 2013). Authors suggested that the loss of function of either *OsVIT1* or *OsVIT2* leads to higher availability of Fe and Zn in flag leaf cells cytoplasm, which would be more readily translocated to seeds. Recently, analyses of a CRISPR-Cas9–generated *OsVIT2* mutant showed that the OsVIT2 protein plays a role in Fe accumulation in the aleurone layer cells and that the loss of function leads to increased Fe accumulation in the endosperm (Zhang et al., 2012; Che et al., 2021). Polished grains from *osvit2* mutant plants cultivated in soil accumulated 1.4–1.8 times more Fe than WT grains without yield reduction (Che et al., 2021), demonstrating that this gene is a target for biofortification.

OsVMT/OsZIFL12 (vacuolar mugineic acid transporter/zinc-induced facilitator-like 12; Ricachenevsky et al., 2011; Che et al., 2019) is a tonoplast-localized transporter of DMA with high expression in upper nodes, peduncle, and rachis at the flowering stage. *osvmt* knockout mutants accumulated around twofold higher Fe and 1.6-fold higher Zn than WT lines, in addition to increased DMA concentration, without yield reduction (Che et al., 2019). These results showed the importance of *OsVMT* to homeostasis of Fe and Zn and suggest the possibility to combine *OsVMT* knockout with other promising candidate genes to increase in Fe and Zn concentrations in endosperm. Together, OsVIT1/2 and OsVMT are yet underexplored promising candidates for biofortification.

OsYSL15 was characterized as the main Fe(III)-DMA transporter from the soil into root cells (Inoue et al., 2009; Lee et al., 2009a) and, therefore, a key protein in strategy II Fe uptake for rice plants. Fe concentrations in seeds in OE-OsYSL15 plants were higher than in WT, whereas the seed Fe concentration in *osysl15* lines was ~85% lower than in WT. Zn concentrations were not changed by overexpression or knockout of *OsYSL15* (Lee et al., 2009a). Another functionally characterized metal transporter of the same gene family is OsYSL2, which localizes to the plasma membrane and transports Fe(II)-NA and manganese Mn(II)-NA into the phloem, making it important for long-distance transport of metals, including transport into the grain (Koike et al., 2004). The Fe concentration in seeds of *osysl2* knockout lines was 37% lower than in WT plants. Strikingly, when *OsYSL2* was overexpressed under the control of *OsSUT1* promoter, a transporter of sucrose from the phloem to seeds, Fe concentration increased up to 4.4-fold higher than that in WT polished seeds (Ishimaru et al., 2010).

OsYSL9 was identified as a plasma membrane Fe(II)-NA and Fe(III)-DMA transporter, which is responsible for Fe transport from endosperm to embryo in developing seed (Senoura et al., 2017). Fe concentration was slightly higher in brown seeds of *OsYSL9-RNAi* plants than in WT seeds, whereas Fe concentration in *OsYSL9-RNAi* embryos was reduced when compared with WT. However, the Fe concentration was more than twofold higher in polished seeds of *OsYSL9-RNAi* than of WT (Senoura et al., 2017). *OsYSL13* is plasma membrane localized, is induced in roots by Fe deficiency, and has higher expression in the leaf blades and sheaths of plants during vegetative, flowering, and grain filling stage (Zhang et al., 2018). OE-*OsYSL13* and *osysl13* knockout lines were evaluated for Fe concentration in brown rice and seeds. The Fe concentration in *osysl13* mutant plants was lower than in WT whereas higher in OE*-OsYSL13* lines (Zhang et al., 2018).

Barley HvYS1 is an Fe(III)-PS transporter, performing a similar role compared with OsYSL15 (Murata et al., 2006). Based on the assumption that constitutive *HvYS1* expression might improve Fe uptake, Banakar et al. (2017a) expressed *HvYS1* driven by *ZmUbi* promoter in rice plants. Fe concentration in polished seeds from plants expressing *HvYS1* (8.7 µg g^-1^) were 2.1-fold higher than in WT plants (average 4.0 µg g^-1^). In addition, transgenic lines also accumulated significantly higher levels of DMA in all tissues evaluated (Banakar et al., 2017a). Additionally, the selective loading of Fe into the endosperm by *HvYS1* make this transporter an interesting target for rice and barley Fe biofortification.

OsMIT, a mitochondrial Fe transporter, is essential for rice growth and development (Bashir et al., 2011). Fe concentration was significantly lower in unpolished and polished seeds from *osmit* knockdown lines than in WT plants. However, polished seeds from *osmit* plants accumulated higher Zn concentrations when compared with WT (Bashir et al., 2013). These data show that changing transporter expression and/or tissue-specific patterns is a promising avenue for biofortification.

## Iron accumulation in rice seeds as a result of genetic modification for gene functional characterization

Studies with other aims, including gene functional characterization, often result in changes in metal concentration, which could then be harnessed for generating biofortified rice plants (**Table 1** and **Figure 1**). A study in rice seedlings based in global gene expression showed that copper (Cu) had an impact on Fe homeostasis (Andrés-Bordería et al., 2017). To investigate this cross talk, the authors developed transgenic lines constitutively expressing *AtCOPT1*, a high-affinity Cu transport protein from *A. thaliana*. The Fe concentrations in the unpolished and polished rice grain were higher than in the WT by 30 and 60%, respectively. Additionally, young leaves from mutant lines had higher Fe concentrations than old leaves in mutant lines, confirming that ectopic expression of *AtCOPT1* in rice plants affects Fe homeostasis in sink tissues (Andrés-Bordería et al., 2017).

Aiming to increase *in planta* Fe accumulation, as Fe acts as a catalyst to enhance the degradation of lignocellulosic biomass for biofuel production, a fusion polypeptide CBM-IBP, (combining carbohydrate-binding module family 11 [CBM11] and an iron-binding peptide [IBP]), was engineered for secretion into rice cell walls. There was an increase of threefold in Fe concentration in polished seeds from engineered lines compared with WT (Yang et al., 2016).

OsRMC is a receptor-like protein associated to the positive regulation of Fe acquisition in rice plants. When transgenic OE-*OsRMC* and knockdown *osrmc* lines were evaluated, Fe concentration in seeds from OE-*OsRMC* plants were approximately 9% higher than in WT. However, in knockdown lines the Fe concentrations were 17% lower than in WT seeds. Additionally, Zn concentrations were also significantly higher in OE-*OsRMC* plants and lower in knockdown lines. These results show that overexpression of *OsRMC* led to enhanced accumulation of Fe and Zn in mature seeds (Yang et al., 2013).

Recently studies demonstrate that high rates of elevated atmospheric CO_2_ concentrations have a significant impact on the growth, development, and yield of rice plants (Kanno et al., 2017). Studies have shown that the concentrations of essential macro- and micronutrients as Ca, Mn Mg, Fe, and Zn are decreased by elevated atmospheric CO_2_ concentrations in rice polished grains (Guo et al., 2015; Al-Hadeethi et al., 2019). A small rice GTPase *OsRab6a* was identified as playing a role on regulation of Fe homeostasis (Yang and Zhang, 2016). Aiming to understand the role of *OsRab6a*, overexpressing *OsRab6a* plants were developed. Transgenic lines produced higher biomass and increased grain yield when cultivated under elevated CO_2_ rates. The expression of genes associated with Fe acquisition was increased in OE*-OsRab6a* plants under elevated CO_2_ rates. Overexpression of *OsRab6a* had an impact on grain Fe concentration, with higher levels of Fe under elevated CO_2_ conditions than in WT plants under the same treatment. These results suggest that *OsRab6a* can potentially mitigate the reduction on nutritional quality and to improve the yield of rice plants under elevated CO_2_ rates (Yang et al., 2020a).

## Zn homeostasis and biofortification: Still lagging behind

Rice grain Zn concentration is also a quantitative trait and is dependent on several processes such as availability of mineral in soil solution, uptake from soil, assimilation within the plant body, and remobilization into the grain (Sperotto et al., 2013; Garcia-Oliveira et al., 2018; Suman et al., 2021). Recently, some significant progress has been made in developing and releasing high Zn rice varieties, with new varieties being released for commercial cultivation in Indonesia, India, Bangladesh, and the Philippines (Inabangan-Asilo et al., 2019; Anusha et al., 2021). However, the capacity to apply basic knowledge of Zn homeostasis to biofortification is still lagging behind compared with Fe. This is highlighted by the fact that most Zn biofortified lines generated were obtained with Fe as a priority. It also demonstrates that our understanding of basic Zn homeostasis is less explored than Fe homeostasis.

Fe and Zn can both be transported as divalent cations by some transporters and seem to share phloem translocation mechanisms to developing seeds (Zhang et al., 2012). However, few studies specifically attempted to increase Zn concentration in rice seeds (**Table 1** and **Figure 1**). Moreover, besides recent advances, functional characterization of genes that resulted in increased Zn concentration are also less common than those related to Fe homeostasis in rice.

The *ZIP* gene family is the most commonly associated with Zn homeostasis in plants. There are 16 members in the rice *ZIP* family. Two of those genes, *OsZIP5* and *OsZIP9*, are duplicated *in tandem* in the rice genome. The proteins encoded by these genes were clearly shown to be the primary Zn transporters from the soil into rice roots (Huang et al., 2020; Tan et al., 2020; Yang et al., 2020b). The knockout of *OsZIP5* and/or *OsZIP9* resulted in lower grain Zn concentration and in significant yield loss under field condition. Likewise, the overexpression of *OsZIP5* resulted in reduced Zn concentration in brown rice and also in yield loss (Lee et al., 2010). On the other hand, the overexpression of *OsZIP9* increased grain Zn concentration in brown rice by approximately 1.3-fold, when compared with WT plants, without yield penalty (Tan et al., 2020). If increased *OsZIP9* expression can result in high Zn concentration in polished grain as well, this gene could have a high potential for rice Zn biofortification.

Two genes encoding metallothioneins (MTs) highly expressed in rice nodes were identified, *OsMT2b* and *OsMT2c*. Both genes play a role on scavenging reactive oxygen species and are associated with stress tolerance (Wong et al., 2004; Steffens and Sauter, 2009; Liu et al., 2015). Knockout lines of *osmt2b*, *osmt2c*, and *osmt2bosmt2c* were evaluated for Zn seed concentration. In brown rice grains, Zn concentration was lower in single and double mutants of *OsMT2b* and *OsMT2c* than in WT, with the double mutant showing a more severe reduction in Zn concentration. However, *osmt2b* and *osmt2c* accumulated more Zn in node I. In addition, decreased grain yield was observed (Lei et al., 2021). These results indicate that the knockout of *OsMT2b* and *OsMT2c* consistently decreased the distribution of Zn to the panicle but increased in the upper nodes and make these genes the targets for Zn biofortified rice using overexpression.

DMAS is a member of the aldo-keto reductase super family, encoding the protein that performs the last step in DMA synthesis. Brown and polished seeds from soil-grown *dmas* knockdown plants accumulated significantly higher Zn levels (1.2-fold) than WT seeds (Bashir et al., 2017). However, no differences in Fe concentration between brown and polished rice was observed. It would be interesting to further explore how the manipulation of the PS biosynthesis pathway and DMA transport could be combined in order to further increase Fe and/or Zn concentrations in polished grains.

## The potential of approaches combining multiple Fe and Zn homeostasis genes

Approaches simultaneously employing multiple Fe and Zn homeostasis genes have the potential to combine the best features of each and be more effective. *OsNAS2* was expressed under the control of *CaMV35S* promoter and soybean *Ferritin* gene (*GmFer-H1*) under the control of *GlutelinA2* (*GluA2*) promoter as a single cassette in variety IR64. Fe concentration in polished seeds was 15 µg g^-1^, an increase of 7.5-fold compared with WT in field conditions in two different sites. Additionally, Zn concentration increased by 2.7- to 3.8-fold WT, achieving up to 53.8 µg g^-1^ (Trijatmiko et al., 2016).

Transgenic lines simultaneously expressing two soybean *Ferritin* (*GmFer-H2*) genes under the control of two endosperm-specific promoters, the *OsGlB1* and *OsGluB1*; barley *HvNAS1* gene under the control of *OsActin1* promoter; and rice *OsYSL2*, an Fe(II)-NA transporter (Koike et al., 2004), under the control of a sucrose transporter promoter (*OsSUT1*) and *OsGlB1* promoter; showed 4.4-fold Fe and 1.6-fold Zn concentration increase when cultivated in the field (Masuda et al., 2012). The same strategy was employed by Aung et al. (2013) using variety Paw San Yin, which naturally has high Fe concentration in polished seeds. The transgenic lines accumulated 3.4- and 1.3-fold higher Fe and Zn concentrations, respectively, in polished seeds compared with WT (Aung et al., 2013).

Another strategy was developed by Masuda et al. (2013). Tsukino Hikari rice cultivar was transformed with constructs containing *GmFerH2*, one driven by *OsGluB1* and another by *OsGlB* endosperm-specific promoters; with barley *HvNAS1*; with two *NAAT* barley genes, *HvNAAT-A*, *HvNAAT-B*; and an MA synthase gene (*HvIDS3*). Authors aimed to combine the results from *ferritin* expression in endosperm while increasing MA concentration in rice plants. The average Fe concentrations in polished seeds ranged from 2.5- to 4-fold higher than WT lines. Additionally, Zn concentrations in polished seeds was, on average, 1.35-fold higher than in the WT (Masuda et al., 2013). This work suggested that ferritin combined with enhanced accumulation of PSs can lead to synergistic effects on biofortification.

Based on the same constructs previously used by Lucca et al. (2001) (and described above), Wirth et al. (2009) introduced in *japonica* cultivar Taipei 309 the *Ferritin* gene from *P. vulgaris* (*PvFer*) and a *phytase* gene from *Aspergillus fumigatus* (*AfPhytase* - to reduce the phytate content and improve Fe bioavailability in rice seeds) both under the control of *OsGlB* promoter. Authors also introduced *A. thaliana NAS* gene (*AtNAS1*) under the control of a constitutive promoter. Fe concentration in polished seeds was 6.3-fold higher than in WT, whereas Zn was 1.3- to1.5-fold (Wirth et al., 2009). Employing the lines developed by Wirth et al. (2009); Boonyaves et al. (2016) further expressed *AtIRT1* driven by *Medicago sativa Early Nodulin 12B* (*MsEnod12B*) promoter, which drives expression in rice vascular tissue and root epidermal cells (Boonyaves et al., 2016). The introduction of *AtIRT1* increased Fe concentration from 4 µg g^-1^ (for plants grown in soil) to 9.6 µg g^-1^ in polished grains of plants grown in soil, a 2.2-fold increase when compared with original lines. In addition, Zn concentrations increased up to 1.5-fold in polished grains when compared with the WT plants (Boonyaves et al., 2016). These results point to the fact that the simultaneous expression of *AtIRT1*, *AtNAS1*, and *PvFer* is an effective strategy to biofortify Fe and Zn in rice. It also highlights the importance of accessing previously generated biofortified lines, allowing for comparison of different combination of transgenes without the need to generate events *de novo*.

Following a similar strategy, different constructs were introduced in rice with two distinct promoters controlling *AtIRT1* expression: *MsEnod12B* or native *AtIRT1* promoter. Each construct was expressed together with *PvFer* driven by *OsGlB1* and *AtNAS1* driven by *CaMV35S* promoters as a single locus gene cassette (Boonyaves et al., 2017). In plants carrying *AtIRT1* under the control of *MsEnod12B* promoter, polished grains had 5.0 µg g^-1^ Fe concentration, whereas the average values observed in control lines were 2.73 µg g^-1^ (a 1.8-fold increase). When *AtIRT1* was expressed under the control of its native promoter, values reached 10.46 µg g^-1^ Fe, which represents a 3.8-fold increase. Furthermore, these values reached 4.7-fold higher than the Fe concentration observed in WT line in T3 polished grains, demonstrating that the phenotype might be stable after multiple generations. Zn concentrations were also significantly increased in polished grain (1.8-fold) from the line expressing *AtIRT1*::*AtIRT1* (Boonyaves et al., 2017).

The manipulation of citrate efflux into the xylem, which binds Fe and allows its transport with the transpiration stream, was also evaluated for biofortification (Durrett et al., 2007; Roschzttardtz et al., 2011). Transgenic rice plants were generated constitutively expressing *AtFRD3* (*Ferric Reductase Defective 3*), which encodes a transporter that loads citrate into the xylem (Durrett et al., 2007) together with *PvFer* controlled by the *OsGlB1* promoter and expressing *AtNAS1* controlled by a constitutive promoter. The rationale was to facilitate long-distance Fe transport as well as efficient Fe uptake and storage in the rice endosperm. Citrate levels in the xylem sap were 2.0-fold higher in transgenic lines than in WT. Overexpressing lines also had increased Fe levels in polished rice grain (3.3- to 5.3-fold), when compared with WT. Zn concentrations in polished grains were also significantly higher (up to 2.44-fold). Furthermore, lines were tolerant to low Fe and to aluminium toxicity (Wu et al., 2018). These results show that manipulating citrate levels through expression of *AtFRD3* or other similar transporters has potential for biofortification.

Similarly, Wu et al. (2019) developed transgenic lines expressing *AtNRAMP3* (*Natural Resistance Associated Macrophage Protein 3*) together with *AtNAS1* and *PvFeR*. AtNRAMP3 is localized to the vacuolar membrane (Thomine et al., 2003) and was expressed under the control of *ZmUbi* or rice embryo/aleurone-specific *18 KDa oleosin* (*OsOle18*) promoters. Fe levels in polished rice grain from all four constructs (*CaMV35S::AtNAS1* + *OsGlB1::PvFer* + *ZmUbi::AtNRAMP3*; *CaMV35S::AtNAS1* + *OsGlB1::PvFer* + *OsOle18::AtNRAMP3*; *OsGlB1::PvFer* + *ZmUbi::AtNRAMP3* and *OsGlB1::PvFer* + *OsOle18::AtNRAMP3*) were 3.6–6.0 times higher than in WT. Zn levels were also increased (1.5- to 2.4-fold) (Wu et al., 2019). Additionally, the authors introduced *AtNRAMP3*, *AtNAS1*, and *PvFer* cassette in an elite variety, IR64, resulting in higher Fe and Zn levels in polished grain of transgenic IR64 lines, achieving more than 90% of the recommended iron increase (and 170% of the Zn recommended target increase) in rice polished grain (Wu et al., 2019).

Recently, novel rice Fe biofortification strategies have been reported (Kawakami et al., 2022). The first strategy involved expression of maize *YS1* controlled by the *OsHMA2* promoter (*OsHMA2*::*ZmYS1*), leading to 4.8- and 1.5-fold increases in Fe and Zn concentrations in polished grains, respectively. The second strategy involved *OsTOM1* expressed under the control of *OsFRDL1* promoter (*OsFRDL1*::*OsTOM1*), leading to 3.2- and 1.2-fold increases in Fe and Zn concentrations in polished grain, respectively. However, when both strategies were combined, no synergistic effects were observed. Authors further introduced a third construct containing the *GmFER* gene under control of endosperm-specific promoter (*OsGlob1*:*GmFER H-1*). The three-gene strategy resulted in Fe and Zn concentrations increased up to 8.7- and 1.4-fold, respectively, compared with the WT. When the three constructs were combined with constitutive expression of *HvNAS1* (*ZmUbq1::HvNAS1*), Fe and Zn concentrations increased up to 9.3- and 1.2-fold. This last strategy showed that constitutive *HvNAS1* expression does not have a strong additive effect on Fe and Zn concentration in polished grain, when compared with the third strategy. Interestingly, the single, two-, and four-gene strategies showed comparable total grain weight to WT, whereas most lines in the three-gene strategy showed decreased total grain weight (Kawakami et al., 2022). This work highlights the variable impact that transgenic approaches can have on yield and suggest that advanced lines should be carefully selected, because some effects might be derived from expression level and cassette insertion position, which could lead to variability between events carrying the same constructs and not only as consequences of transgene expression on physiology.

## Concluding remarks and future directions

Although the field of biofortification has made progress in testing multiple strategies and achieved relative success in increasing Fe and Zn in rice grain and endosperm, we still do not have consistent, reliable, and productive elite genotypes with target concentrations being cultivated for human consumption. To deliver that to the public, research in the field might need to focus on issues not addressed yet.

There is a clear need to improve our basic understanding of metal transport to seeds in rice and other model species. Work in the model species *A. thaliana* showed that Zn loading in developing seeds from the mother plant is performed by AtHMA2/AtHMA4 transporters (Olsen et al., 2016), raising the question whether rice OsHMA2, which was already shown to function similarly to the *A. thaliana* orthologs in roots (Takahashi et al., 2012), can have a similar function. However, it is important to consider the differences in seed development and structure in eudicots and Poaceae monocots, especially the mature seed endosperm (Galland et al., 2017). Recent work has characterized OsVIT2, which is involved in Fe loading in rice grain (Che et al., 2021), and OsZIP4, which is important for Zn transport to developing reproductive tissues (Mu et al., 2021). OsVIT2 is from the same gene family as AtVIT1, but their functions are not the same regarding how they determine Fe localization in seeds, given the distinct Fe distribution in each species. Moreover, the nodes have been shown to be crucial in controlling metal transport to reproductive tissues in rice (Yamaji and Ma, 2017), highlighting some limitations of using *A. thaliana* as a model. In that regard, attempts to carry out biofortification in other closely related plant species, such as wheat (Sheraz et al., 2021; Kong et al., 2022), might benefit the rice biofortification field, informing new strategies that could work in rice as well. Still, rice is the model species for cereals, and research on basic Fe and Zn homeostasis in rice is likely to provide the best strategies for biofortification in cereals in general.

Another important problem that should be addressed is the adequate identification of biofortification approaches that do not compromise yield. Kawakami and Bhullar (2021) have summarized which lines maintain and which have decreased yield-related phenotypes. However, this information is not always present in biofortification studies. Moving forward, as different approaches and combinations are attempted, successful events are introduced in local genetic backgrounds, and trials in specific growth conditions are performed, it will be important to compare different studies. One possibility is the adoption of metadata standards, such as those proposed for RNA sequencing (Kumar et al., 2015), microbiome in agriculture (Dundore-Arias et al., 2020), or field trials (Grosse et al., 2021; Walters et al., 2021). At a minimum, reporting on growth, yield and providing open access raw data would improve our ability to evaluate and compare the most successful constructs, events, and combinations of transgenes/mutants.

Thus far, the biofortification field has relied much on transgene insertion for changing nutrient concentration in seeds. However, transgenics are still likely to suffer major resistance from the public, regardless of adequate safety testing. Therefore, a promising strategy could be the identification of mutations, which increase Fe and Zn concentration in seeds and that could be generated by genome editing technologies. For example, CRISPR-Cas9–based loss of function of *OsVMT* (Che et al., 2019) or *OsVIT2* (Che et al., 2021) increased Fe (and Zn in *osvmt* mutants) concentration in rice endosperm without yield penalties. Identifying other loss-of-function mutations as well as regulatory regions that can be changed by CRISPR-based gene editing and that result in target phenotypes could lead to biofortified rice without transgenes. Pyramiding combinations of such mutations would be feasible to deliver non-transgenic, Fe and Zn-enriched grains to the consumer. Moreover, such initiatives could benefit from better data and materials (such as seeds) sharing among those interested in the field, allowing for statistical and experimental comparisons between lines generated by different groups in variable experimental settings. These practices could accelerate the delivery of biofortified rice to the public.

## Author contributions

AW, FR, and SL conceived and initiated the review and were involved in drafting and critically revising the manuscript. All authors contributed to the article and approved the submitted version.

## Funding

SL was supported by the Institute for Basic Science (IBS-R013-D1); FR was funded by CNPq (Conselho Nacional de Desenvolvimento Científico e Tecnológico, Brazil) and FAPERGS (Fundação de Amparo à Pesquisa do Estado do Rio Grande do Sul, Brazil). AW funding is currently being provided by the Alexander von Humboldt Foundation (Germany).

## Acknowledgments

We would like to thank Dr. Janette P. Fett for proofreading and commenting on the manuscript.

## Conflict of interest

The authors declare that the research was conducted in the absence of any commercial or financial relationships that could be construed as a potential conflict of interest.

## Publisher’s note

All claims expressed in this article are solely those of the authors and do not necessarily represent those of their affiliated organizations, or those of the publisher, the editors and the reviewers. Any product that may be evaluated in this article, or claim that may be made by its manufacturer, is not guaranteed or endorsed by the publisher.
